# RNA virus diversity highlights the potential biosecurity threat posed by Antarctic krill

**DOI:** 10.1007/s42995-024-00270-w

**Published:** 2025-02-13

**Authors:** Tingting Xu, Xianyong Zhao, Thomas Loch, Jiancheng Zhu, Wei Wang, Xinliang Wang, Chong Wang, Gangzhou Fan, Bin Hao, Jichang Zhang, Wenxiu Zhao, Melba G. Bondad-Reantaso, Victoria Alday-Sanz, Qingli Zhang

**Affiliations:** 1 State Key Laboratory of Mariculture Biobreeding and Sustainable Goods; Key Laboratory of Maricultural Organism Disease Control, Ministry of AgricultureQingdao Key Laboratory of Mariculture Epidemiology and BiosecurityYellow Sea Fisheries Research Institute, Chinese Academy of Fishery Sciences, Qingdao, 266071 China; 2 Laboratory for Marine Fisheries Science and Food Production Processes, Laoshan Laboratory, Qingdao, 266237 China; 3 Aquatic Animal Health Laboratory, Michigan State University, East Lansing, MI 48824 USA; 4 Fisheries and Aquaculture Division, Food and Agriculture Organization of the United Nations (FAO), 00153 Rome, Italy; 5 Breeding Programs and Research and Development National Aquaculture Group (NAQUA), Jeddah, 21541 Kingdom of Saudi Arabia

**Keywords:** RNA virome, Antarctic krill *Euphausia superba*, Biosafety, Global aquaculture, Antarctic ecosystem

## Abstract

Antarctic krill *Euphausia superba*, one of the most abundant species on the planet, is a keystone species of the Southern Ocean ecosystem. In the present study, we analyzed the RNA virome of Antarctic krill via metatranscription methods. The results showed that only 0.39% (49/12, 558) of the resultant unigenes could be assigned to known viral taxa, which were most similar to 17 known viruses, including nine invertebrate viruses, two vertebrate viruses, three protozoan viruses and three mycoviruses. However, most of the detected viruses possessed low amino acid similarity with counterparts in the viral databases. *Penaeus vannamei* picornavirus (PvPV; Family Picornaviridae) and covert mortality nodavirus (CMNV; Family Nodaviridae) were the two most abundant viruses in the Antarctic krill RNA virome. Notably, PvPV and CMNV are known pathogens to multiple aquatic animals according to epidemiological survey and exposure experiments, whereby PvPV positive krill caused clinical symptoms and histopathological lesions to *P. vannamei* and similarly, CMNV infection altered the swimming and feeding behavior of parent marine medaka *Oryzias melastigma* and caused tissue damage and even spinal curvature of the offspring. Results herein reveal, for the first time, the high abundance and taxonomic diversity of viruses in Antarctic krill while simultaneously highlighting the risk of an important virus reservoir to global aquaculture, and the potential impact on animals in the Antarctic ecosystem.

**Supplementary Information:**

The online version contains supplementary material available at 10.1007/s42995-024-00270-w.

## Introduction

Viruses, which are abundant and ubiquitous in the environment, not only affect the genetic diversity and community structure of microbes (Mann et al. 2003; Rodriguez-Valera et al. 2009; Schulz et al. 2020; Thompson et al. 2024), but also influence the evolution of their hosts, food web structures, and population dynamics of globally dominant species (Ignacio-Espinoza et al. 2020; Suttle 2005; Van Etten 2011). Marine viruses exert a strong and ongoing impact on marine ecology, affecting ecosystem stability (Fischer et al. 2010; Lima-Mendez et al. 2015). Indeed, a series of successive publications revealed that marine viral communities are passively transported by oceanic currents and are an important driver of global geochemical cycles and the evolution of marine ecosystems (Brum et al. 2015; El-Sayed and Kamel 2021; Gregory et al. 2019; Lima-Mendez et al. 2015; Miner et al. 2021; Roux et al. 2016; Welsh et al. 2020).

Antarctica is an ice-covered landmass that was isolated from the rest of the world for over 20 million years (Zachos et al. 2001). It was once thought to be a sterile or relatively “uncontaminated” environment due to its extreme climate and isolated geographical location (Chown and Convey 2007; Convey and Smith 2010; Lee et al. 2016), but substantial microbial diversity, particularly bacteria, are now known to be present in Antarctica (Chown et al. 2015; Lambrechts et al. 2019). However, knowledge of the viruses present in Antarctica was incomplete due to the limitations of traditional methods for virus detection and characterization. Concerns about the thawing of ancient polar ice, which could release active viruses preserved for thousands and millions of years, are attracting increasing attention as climate change becomes one of the most pressing global challenges (El-Sayed and Kamel 2021; Miner et al. 2021). To this end, more and more viruses are being identified from Antarctic water, soil and plants thanks to the initiation of viral ‘omics’ studies (Bueno et al. 2009; Cao et al. 2020; Cavicchioli and Erdmann 2015; Daniel et al. 2016; Gregory et al. 2019; Lopez- Varsani et al. 2017; Smeele et al. 2018; Yau and Seth-Pasricha 2019).

Antarctic krill (*Euphausia superba*), a shrimp-like crustacean, has an estimated abundance of 300–500 million tons (Sun and Liu 2009; Sylvester et al. 2021), thereby representing the largest biomass of multicellular wild animal species on the planet (Meyer et al. 2020). These krill play the central role in the Southern Ocean, serving as a link between primary production and upper trophic level predators, such as fish, whales, penguins, seals and seabirds (Meyer et al. 2020; Quetin et al. 2007; Sun and Liu 2009). Indeed, Antarctic krill is a keystone species in the Antarctic ecosystem, and its commercial value has been increasing since the 1970s (Sun and Liu 2009), mainly for human consumption and as feed for aquaculture systems (Yoshitomi and Nagano 2012). Given the key role of Antarctic krill in the Antarctic food web and its high value in commercial fisheries, numerous studies have been conducted in recent decades on its population dynamics, life cycle, ethology and nutritional content (Cavan et al. 2019; Meyer et al. 2020; Wille et al. 2020).

Despite this importance, relatively little is presently known about viral diversity in Antarctic krill, as well as the concomitant risk of exposure to such viruses by organisms that feed upon them. To this end, we investigated the RNA virome of Antarctic krill using metatranscription methods, also assessing the pathogenicity of the two most abundant krill viruses to farmed aquatic animals and their potential risks.

## Materials and methods

### Sample collection

**Fig. 1:**
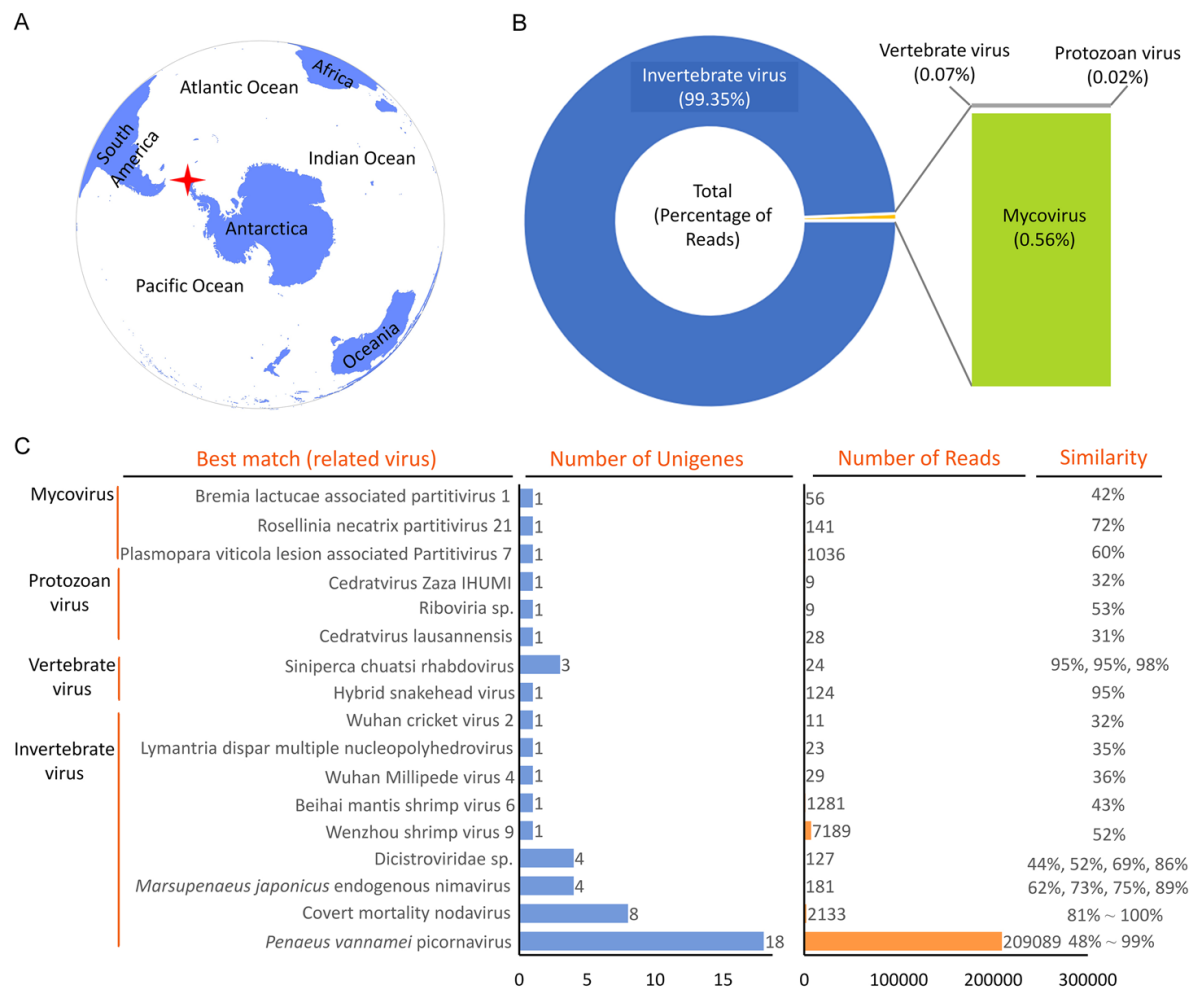
Overview of the sampling site and the RNA virome data. **A** World geographical map showing the geolocation of the Antarctica pole. The red asterisk represents the sampling site. **B** Percentage of viruses in different host groups from Antarctic krill. (C) The number and the similarity of reads and unigenes related to known viruses (GenBank accession numbers listed in Supplementary Table S2)

Krill samples for metatranscription analyses were collected on board the Chinese krill fishing vessel FU RONG HAI, which fished in Antarctic Peninsula waters in July 2015 (Fig. 1A). Sampling depths ranged from 60 to 130 m. Samples were stored at − 20 °C on board, and then transferred to − 80 °C in the laboratory. Samples used to investigate the prevalence of PvPV and CMNV in Antarctic krill were collected from sub-area 48.1 in April–May 2017, from CCAMLR Sub-areas 48.1, 48.2 and Division 58.4.2 in January 2018, and from sub-area 48.2 in January 2019, respectively. Four to six individual Antarctic krill were randomly taken from each sampling site for analysis. The whole individual in each sample was divided equally into two aliquots along the longitudinal axis and the cephalothorax of one of the two aliquots was preserved in 4% paraformaldehyde (4% PFA) solution (Sinopharm, Beijing, China). The second aliquot was then cut into two parts and preserved in RNAstore solution (Tiangen, Beijing, China) and 2.5% glutaraldehyde solution (Sinopharm, Beijing, China), respectively. The remaining part of each individual was homogenized and smeared onto Whatman FTA Elute cards (GE Healthcare Life Sciences, Marlborough, MA, USA). Each FTA Elute card was air dried separately for 30 min, then placed in an independent plastic bag, sealed and stored at − 20 °C. Nine individuals of *Electrona carlsbergi* and *Mesonychoteuthis hamiltoni* samples were collected from Sub-area 48.2 in January 2018. The eyes of each individual were removed and stored in RNAstore solution for molecular biological detection.

### Virus extraction

Seven randomly taken frozen krill (body length 3–5 cm) from different sampling sites were thoroughly homogenized in TN buffer (20 mmol/L Tris/HCl, 400 mmol/L NaCl, pH 7.4) on ice. The interstitial fluid of the homogenate was then centrifuged at 1400 *g* for 15 min at 4 °C, after which the supernatant was centrifuged for a second time at 10,000 *g* for 25 min at 4 °C. The final supernatant was then centrifuged at 160,000 *g* for 4 h at 4 °C. Finally, the virus pellet was resuspended in TN buffer and stored at − 80 °C for viral-RNA isolation.

### Viral-RNA isolation

Total RNA was extracted from 140 μL of the centrifuged viral crude extract using the Viral Genome RNA Extraction Kit (Tiangen, Beijing, China) according to the manufacturer’s instructions. The quantity and quality of the purified RNA were then measured using the Nanodrop 2000 (Thermo Scientific, Waltham, MA, USA). The final concentrations of the viral-RNA extracts were adjusted to 100–200 ng/µL, and then used for metatranscription analysis, and as a template for genomic cloning of PvPV and CMNV.

### Next-generation sequencing and bioinformatics analysis

Firstly, rRNA was removed using the Ribo-ZeroTM Magnetic Kit (Epicentre, Madison, WI, USA) to enrich for RNA. Secondly, the enriched RNA was fragmented into small pieces and reverse transcribed into the cDNA using random primers. Thirdly, the cDNA fragments were purified, ends repaired, one base added, and ligated to Illumina sequencing adapters. Finally, the size of the ligation product was selected by agarose gel electrophoresis, PCR amplification, and sequencing on the Illumina novaseq 6000 system from Gene Denovo Biotechnology (Guangzhou, China). Raw reads were filtered by removing the linker and low-quality reads containing more than 10% of unknown nucleotides (*N*) and 50% of low-quality (*Q* value ≤ 20) bases, respectively. The clean reads were then de novo assembled into unigenes using the “Trinity” tool and BLASTX against the NCBI GenBank non-redundant (nr) virus database using BLASTX with an E-value cutoff of 10^–5^ (Grabherr et al. 2011; Pruitt et al. 2007). The BLAST results were analyzed to save the best hits for each sequence. The best-hit sequences were individually annotated for taxonomic assignment.

### Reverse transcription loop-mediated isothermal amplification (RT-LAMP) analyses

The discs punched out from the FTA® cards were used for nucleic acids preparation. 1000 μL FTA wash buffer was added to the discs in a microtube and vortexed for three min to wash the discs. These were then transferred into microtubes containing 40 μL TE buffer and incubated at 95 °C five min to denature the RNA. The denatured nucleic acids in the discs were used as the template for the RT-LAMP assay. The CMNV RT-LAMP method was based on the previous report (Zhang et al. 2017). Fluorescence development of the RT-LAMP product was conducted by mixing the pre-sealed GeneFinder™ in the cap of the reaction tube with the reaction mixture according to our previously reported procedure, which was integrated with anti-contamination measures (Li et al. 2019a, b).

### Reverse transcription-nested PCR (RT-nPCR) analyses

Total RNA used for RT-nPCR and RT-qPCR was extracted from RNAstore solution preserved tissues (30 mg) using an RNAprep pure Tissues Kit (Tiangen). The CMNV RT-nPCR was performed according to our previous report with a slight modification (Wang et al. 2021). The first step was conducted using a PrimeScript One Step RT-PCR Kit (TaKaRa). The amplification program was performed at 50 °C for 30 min, followed by 32 cycles of 94 °C for 30 s, 52 °C for 30 s and 72 °C for 30 s, and a final extension at 72 °C for 5 min. The second step was prepared using a TaKaRa Ex Taq kit. The amplification was performed with the following cycling parameters: initial denaturation at 94 °C for 4 min, followed by 30 cycles of 94 °C for 20 s, 50 °C for 20 s and 72 °C for 20 s, and a final extension at 72 °C for 5 min. A 619 bp and 413 bp amplicon was amplified by the first and second step of the PCR, respectively.

### TaqMan real-time reverse transcription quantitative PCR (RT-qPCR) analyses

CMNV RT-qPCR primers, reaction mixture and procedures were referred to the previous report (Wang et al. 2022). The PvPV RT-qPCR was reaction program was determined as reverse transcription at 50 °C for 15 min and denaturation at 94 °C for 5 min, followed by 40 cycles including denaturation at 94 °C for 10 s, and annealing and extension at 60 °C for 30 s. The reaction mixtures were prepared using a TaKaRa One Step PrimeScript™ RT-PCR Kit with the primer sets of PvPV-TAQ-F/R (PvPV-TAQ-F: 5′-ACAGAGAAGCAATCGTTACCC-3′, PvPV-TAQ-R: 5′-AGTGTGATGTCGTGRGTRTG-3′) and the probe of PvPV-TAQ-P (5′-AGTTTTGTGAGGTCAGGGCAGCTAC-3′).

### Histopathological and ISH analyses

For each sample, three paraffin sections (3 μm) were prepared for further histological and in situ hybridization (ISH) analysis according to the reported protocol (Wang et al. 2021). One section was stained with conventional HE using standard methods. One of the two corresponding unstained sections was subjected to ISH assay, and the other section was hybridized with a non-targeted random probe (*Homo sapiens* interleukin 18 (IL18): 5′-CTACTGCAG AAGATGGCTGCTGAACCAGT-3′) as a negative control.

### Cloning of PvPV and CMNV genomes

The PCR primers, reaction mixture and procedures for cloning CMNV and PvPV genomes by PCR were as descripted in the previous report (Liu et al. 2021; Xu et al. 2021). The PCR products were purified using a PCR purification kit (Tiangen, Beijing, China) and then sequenced by the commercial sequencing company of Shanghai SANGAN Co. Ltd (China). Sequence analysis was performed using BioEdit (Ver. 7.1.3) and BLAST (http://www.ncbi.nlm.nih.gov/gorf/). The phylogenetic tree was generated using the neighbor-joining method of MEGA 7.0 (Tamura et al. 2011).

### Assessment of PvPV virulence in white leg shrimp (*P. vannamei*)

Crude extract of PvPV was purified from PvPV-positive Antarctic krill samples according to previously described methods (Liu et al. 2021). The specific pathogen-free (SPF) white-leg shrimp (post-larvae, 6–8 mm in length) were collected from the BLUMP Shrimp Breeding base in Weifang, China. Before challenge, 10 randomly taken white-leg shrimp were collected for PvPV diagnosis by RT-qPCR and ISH, and the results were all negative. White-leg shrimp for virus challenge experiments were cultured in glass tanks containing 800 mL of seawater (at 26 °C, salinity 26 ± 2 g/L) before use. For the exposure experiments, native whiteleg shrimp (0.1 g mean weight) were divided into an immersion infection group and a control group. Each group included three replicates, and each replicate contained 15 individuals. 1 mL of crude PvPV virus extract (5.40 × 10^6^ viral copies/µL) was added to the immersion infection group and maintained for 30 min. The control group received 1 mL of TN buffer, and was likewise maintained for 30 min. The status and death of individuals in the PvPV infection and control groups were then observed and recorded every four hours for a total of 120 h. At the end of the exposure experiment, live shrimp were collected for ISH analysis, and dead shrimp were frozen and stored at −80 ℃. The experimental process may be seen in Fig. 3A.

### Challenge test for potential risk analysis of CMNV in Antarctic krill to its predators

SPF marine medaka (2 months old, sexually immature, mean body length 1.5–2 cm) were purchased from the Guangdong Laboratory Animals Monitoring Institute. The fish were maintained in 30‰ seawater (volume of 80 dm^3^) at 25 ± 1 °C in a 14 h light:10 h dark cycle with constant aeration, feed three times daily and change fresh sea water once daily. Prior to the challenge experiment, 10 marine medaka were randomly taken for CMNV diagnosis by RT-qPCR according to the published methods (Li et al. 2018); the results were all negative.

The CMNV suspension used for the intraperitoneal injection was purified from only CMNV-positive Antarctic krill according to the optimal protocol described in the section dealing with virus isolation. After resuspension in TN buffer, the final pellet was layered onto a 20–50% (w/w) sucrose gradient and centrifuged at 160,000 *g* for 3 h.

In the challenge experiments, marine medaka individuals were divided into four groups, the virus infection groups fed with only CMNV positive Antarctic krill (*per os* infection group), the virus infection groups involving intraperitoneal injection of CMNV suspension (injection infection group), the *per os* infection control groups and the injection infection control groups.

Four replicates were set up for both the *per os* infection and control groups. For the *per os* infection test, one of the four replicates was used for sampling; each replicate included 16 individual fish (eight females, eight males). For the *per os* infection test, each individual in the *per os* infection group and control group was fed Antarctic krill or commercial diets three times a day at a level equivalent to one-tenth of the fish’s body weight. For the injection infection test, each individual in the injection infection group and control group was intraperitoneally injected with 10 µL CMNV virus suspension (1.70 × 10^4^ viral copies/µL) or 10 µL TN buffer, respectively. The status and behavior of the fish were observed and recorded daily.

Prior to sampling, marine medaka were euthanized with excess tricaine methanesulfonate (Sigma, St. Louis, USA) according to a previous protocol (Wilson et al. 2009). To monitor and track changes in the viral load of CMNV in fish, both in the *per os* infection group and its control group, samples (*n* = 2) fish were collected every two days for 14 days post-infection. The head of the marine medaka was collected, and preserved in RNAstore solution for the RT-qPCR assay. On the 15th day post challenge, various tissues of the remaining two fish in the sampling group were collected, and stored in 4% paraformaldehyde (PFA) for histopathological and ISH analysis. Individual fish in the other three replicates of both the *per os* infection group and its control group (no sampling before spawning and hatching) began spawning on the 25th and 34th day after virus infection. Fertilized eggs were transferred to new seawater tanks for hatching. larvae and juvenile fish were observed and recorded daily, and then sampled for histopathology, ISH and RT-nPCR analysis. Body tissues from various parts of each individual in the injection infection group were sampled on the 14th day after challenge. The experimental process of the *per os* infection group and the injection infection group may be referred to seen in Figs. 4A and 5A, respectively.

## Results

### Overview of the RNA virome data sets

A total of 112,773,412 raw reads were generated using the Illumina Hiseq 2500. After quality trimming and filtering, 101,464 (0.09% of raw reads) adapters and 12,179,692 (10.80% of raw reads) low quality reads were removed, leaving 100,492,256 clean reads (89.11% of raw reads) that were retained (Supplementary Table S1). These were then assembled into unigenes. A total of 12,558 unigenes were generated with an average length of 357 bp (Supplementary Table S1).

### Taxonomic profile of the Antarctic krill RNA virome

The above database analysis revealed that, among all the reads, only 0.22% could be assigned to known viral taxa (Supplementary Table S1). These originated from different viral groups associated with distinct hosts: those associated with invertebrates (99.35%), vertebrates (0.07%), protozoans (0.02%) and fungi (0.56%; Fig. 1B). To determine the identity of viruses that were detected in the Antarctic krill RNA virome, an in-depth examination of the metatranscription data was performed using the NCBI online tool BLASTX. A total of 49 unigenes in the Antarctic krill RNA virome were most similar to 17 known viruses, including nine invertebrate viruses, two vertebrate viruses, three protozoan viruses and three mycoviruses (Fig. 1C). Most of the RNA virome unigenes were related to invertebrate viruses, with PvPV being the most abundant, followed by CMNV (Fig. 1C). Among the invertebrate virus-related unigenes, 16 unigenes (ranging from 220 to 2228 bp) had high amino acid similarities (ranging from 80.00 to 99.00%) with the hypothetical proteins of PvPV (Supplementary Fig. S1). A total of eight unigenes (ranging from 203 to 1293 bp) shared high amino acid similarities (ranging from 81.37 to 100%) with amino acid sequences of the CMNV RNA-dependent RNA polymerase (RdRp) and capsid protein (Supplementary Fig. S1). On the other hand, the other invertebrate virus-related unigenes shared low similarities at the amino acid level with their counterparts in the database (Fig. 1C). Among the vertebrate virus-related unigenes, four showed high amino acid homology (95–98%) to known viral pathogens of fish, including hybrid snakehead virus (HSHRV) and *Siniperca chuatsi* rhabdovirus (SCRV; Fig. 1C). Both protozoan virus-related and mycovirus-related unigenes had lower amino acid similarities (31–72%) with known viruses in the NCBI GenBank non-redundant (nr) database (Fig. 1C).

### Prevalence of PvPV and CMNV in Antarctic krill

The prevalence of PvPV and CMNV, the most abundant viruses identified from the Antarctic krill RNA virome, was assessed through a systematic epidemiological survey conducted on Antarctic krill for three consecutive years from 2017 to 2019.

**Fig. 2:**
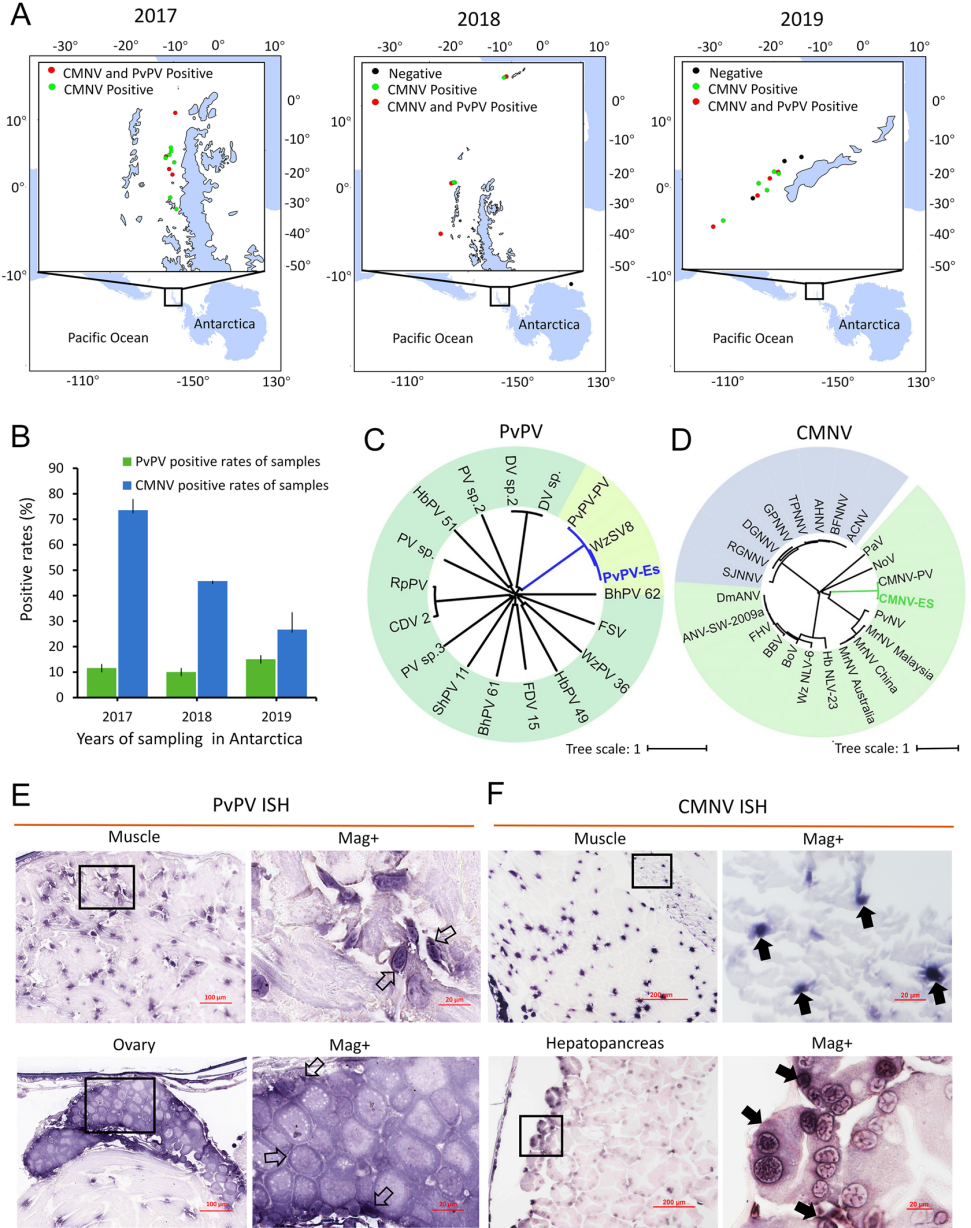
Investigation of the prevalence of CMNV in the Antarctic krill of Antarctica **A** The sampling sites (indicated by red, black and green spots) and the general view of the prevalence scope of CMNV in Antarctica (2017–2019). The black solid spots indicate the sites where Antarctic krill were collected, but no PvPV and CMNV positive samples were identified. The green solid spots indicate sites where only CMNV-positive Antarctic krill were collected. The red solid spots indicate sites where both PvPV and CMNV-positive Antarctic krill were collected. **B** PvPV and CMNV prevalence of the Antarctic krill collected during Antarctica survey (2017–2019). **C** Phylogenetic tree based on the deduced amino acid sequences of the hypothetical protein from PvPV isolates and other picornaviruses (see Supplementary Table S3 for virus abbreviations and GenBank accession numbers). **D** Phylogenetic tree based on the deduced amino acid sequences of the RNA-dependent RNA polymerase (RdRp) from different CMNV isolates and other nodaviruses (see Supplementary Table S3 for virus abbreviations and GenBank numbers). The phylogenetic tree was constructed using the neighbor-joining method with the program MEGA 7.0 and the scale bar was 1. **E** Micrographs of in situ hybridization (ISH) staining for muscle and ovary of the Antarctic krill naturally infected with PvPV. Scale bar = 100 µm and 20 μm for low and high magnification, respectively. **F** Micrographs of ISH staining for muscle and hepatopancreas of the Antarctic krill naturally infected with CMNV. Scale bars = 200 µm and 20 μm for low and high magnification, respectively. The right figure was the magnified micrograph of zone in the black frame in left figure. ISH: in situ hybridization; Mag+ : magnification

A total of 53, 50 and 60 Antarctic krill samples were collected from 11, nine, and 12 sampling sites in one of the main fishing grounds in the Antarctic Peninsula region during 2017–2019, respectively (Fig. 2A). The prevalence of PvPV at the sampling sites from 2017 to 2019 was observed to be 36.67%, 32.5%, and 58.55%, respectively, as determined by RT-qPCR analysis (Fig. 2A). Similarly, RT-LAMP analysis revealed that the prevalence of CMNV at these sites was found to be 100%, 80%, and 75% respectively (Fig. 2A). Furthermore, the prevalence of PvPV and CMNV in the samples was 11.56%, 10.00%, 15.00% and 73.51%, 45.71%, 26.67%, in 2017—2019, respectively (Fig. 2B). Phylogenetic analysis based on the deduced amino acid sequences indicated that the PvPV and CMNV detected in Antarctic krill were closely related to the corresponding sequences of the original isolates that were previously identified from farmed shrimp (Fig. 2C, D). In addition, intense purple hybridization signals of the PvPV and CMNV probes were both observed in the muscle of infected individuals (Fig. 2E, F) via ISH assays. Moreover, the positive hybridization signal for PvPV and CMNV were also detected in the oocytes of the ovary (Fig. 2E) and in the hepatopancreatic tubules (Fig. 2F). No positive hybridization signals appeared on the sections from the same samples with the non-targeted random probes in the hybridization process (Supplementary Fig. S2).

### Assessment of PvPV virulence in white leg shrimp

**Fig. 3:**
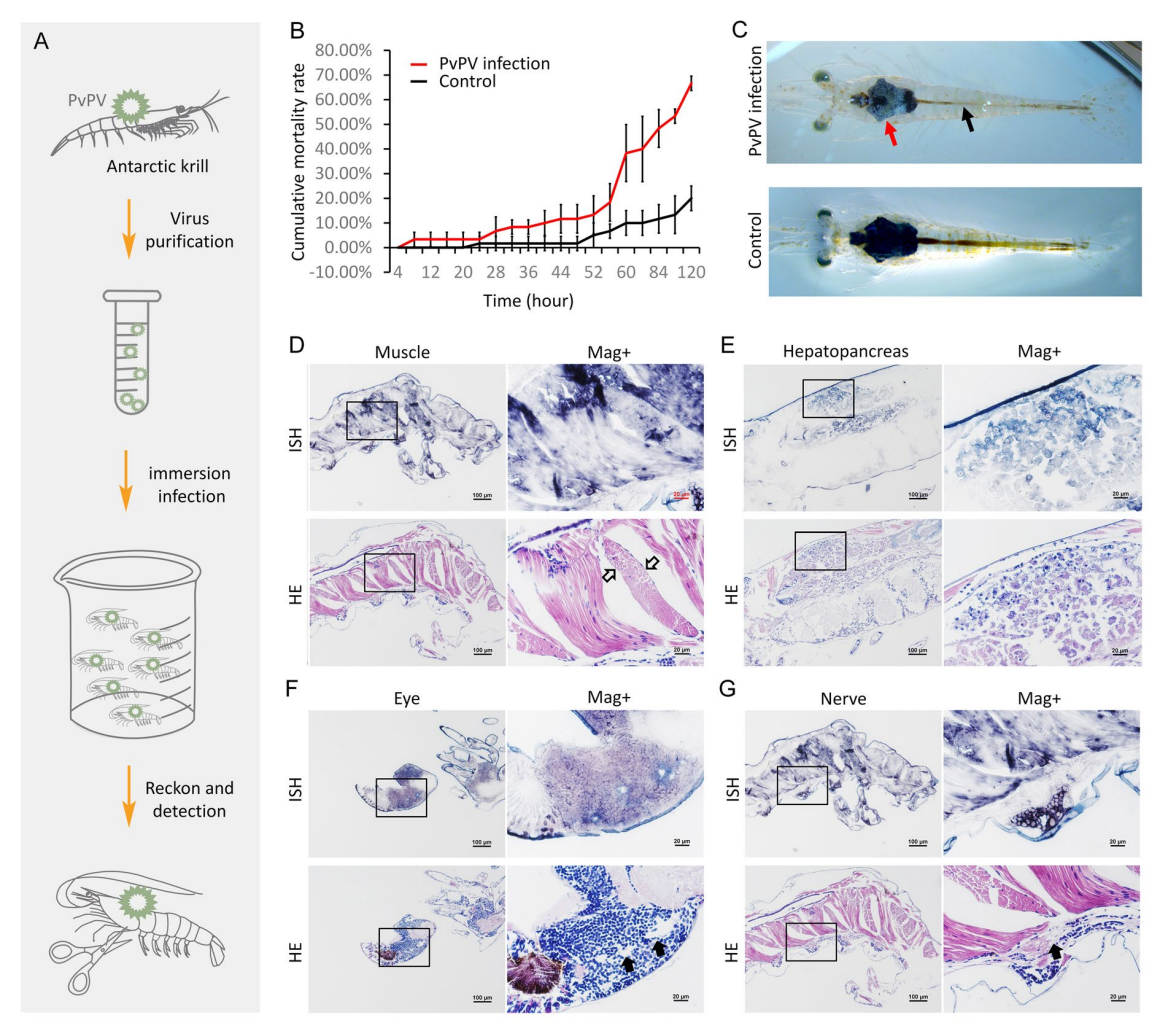
The result of artificial challenge experiment of PvPV infecting *Penaeus vannamei* **A** Schematic diagram of the experimental procedure. **B** Statistical analysis of mortality in the artificial challenge experiment of *P. vannamei*. Cumulative mortality of *P. vannamei* was presented as the mean of data from three replicates for each experimental group (each replicate included 15 individuals). **C** Clinical signs of *P. vannamei* infection by PvPV. The red arrow points the whitening of the connective tissue sheath of the hepatopancreas and shrinkage of hepatopancreas in infected individuals. The black arrow points to the under-filling of the stomach and intestine in the infected individual. Micrographs of in situ hybridization (ISH) and HE staining for **D** muscle, **E** hepatopancreas, **F** eye, **G** nerve of the *P. vannamei* artificially infected with PvPV. Scale bars = 100 µm and 20 μm for low and high magnifications, respectively. The right figure is the magnified micrograph of the zone in the black frame in the left figure. ISH: in situ hybridization; HE: H&E stained; Mag+ : magnification. Black hollow arrow: muscle lysis; Black solid arrow: vacuolation

PvPV was the most abundant virus detected in the Antarctic krill RNA virome, whereby natural infection in Antarctic krill was also proven in the 2017–2019 Antarctic epidemiological investigation. Based on these results, an exposure experiment was conducted to elucidate the virulence of PvPV from Antarctic krill. In PvPV-exposed *P. vannamei,* the cumulative percent mortality reached 66.7% at 120 h after immersion challenge (Fig. 3B). In contrast, the cumulative percent mortality in the mock-exposed *P. vannamei* was 20.00% (Fig. 3B). In PvPV-exposed shrimp, obvious clinical symptoms were observed, including whitening of the connective tissue sheath of the hepatopancreas, atrophy of the hepatopancreas, and a reduction in gastrointestinal contents. In contrast, mock-exposed shrimp did not show any abnormal symptoms (Fig. 3C).

Histopathological changes were observed in moribund shrimp exposed to PvPV, including necrosis of muscle fibers within the abdominal segment (Fig. 3D), lysis of hepatopancreatic tubular epithelial cells, and deformation of hepatopancreatic tubules (Fig. 3E). In addition, severe vacuolization in the globuli cell zone of the ommatidia and the abdominal ganglion (Fig. 3F, G) were evident. Correspondingly, ISH revealed intense positive signals for PvPV in the tissues with the aforementioned lesions (Fig. 3D–G).

### Potential risk of viruses in Antarctic krill to predators

Nine individuals of *E. carlsbergi* and *M. hamiltoni* samples, which are natural predators of Antarctic krill, were collected from the Southern Ocean in 2018. Thirty-three percent (3/9) of the samples (one squid sample and two fish samples) were found to be CMNV positive via RT-qPCR (Supplementary Table S4).

**Fig. 4:**
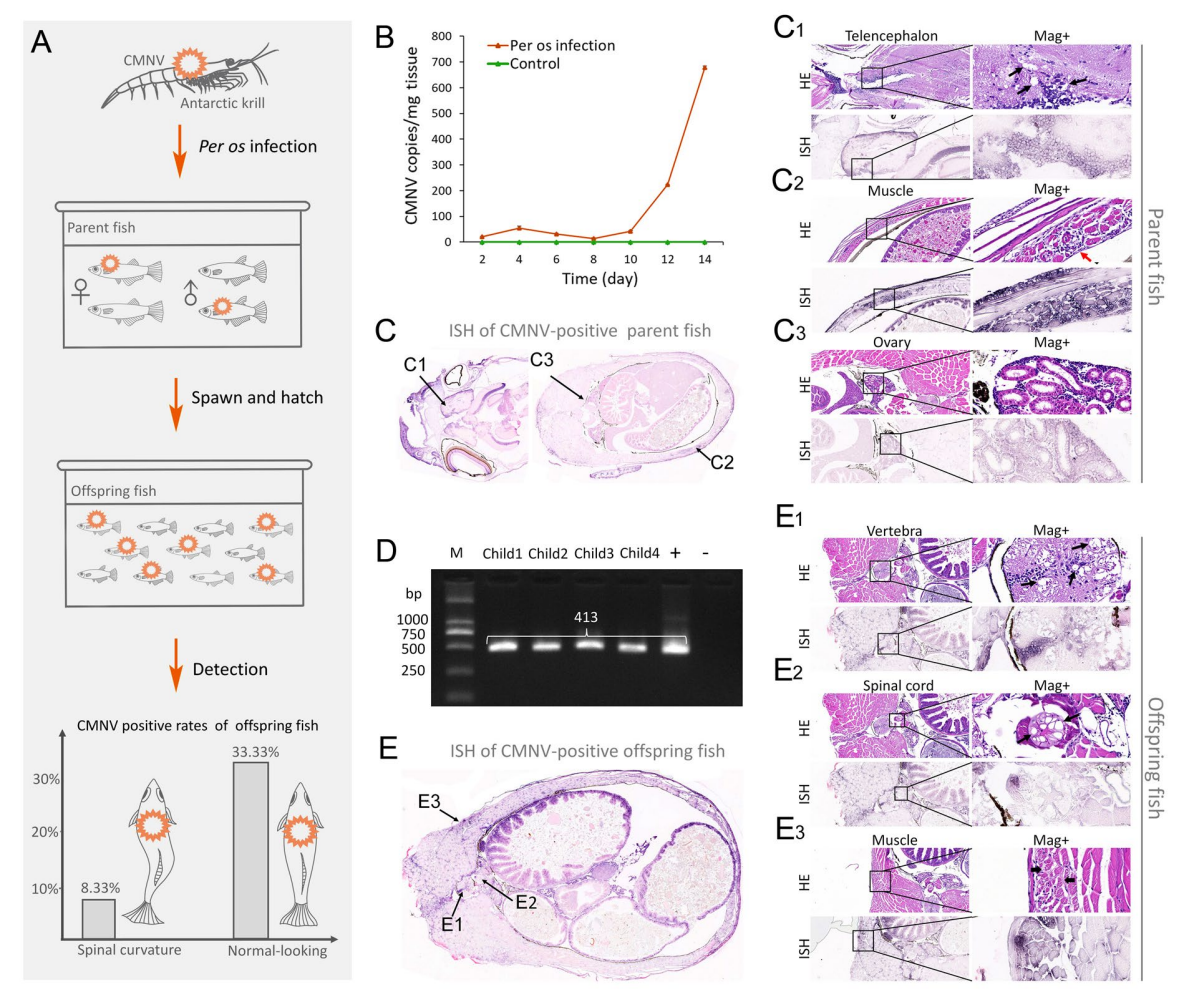
The schema and results of feeding Antarctic krill to marine medaka *Oryzias melastigma* **A** Schematic diagram of the experimental procedure. **B** Changes in CMNV viral load in marine medaka from both the virally infected group fed with CMNV_Es positive Antarctic krill and the control group. *N* = 2. **C** H&E and ISH analysis of marine medaka parents from the viral infection group fed with CMNV_Es positive Antarctic krill. C1–C3 indicate the positions of the telencephalon, muscle and ovary, respectively. (C1) Micrographs of HE staining and ISH for telencephalon. Note the vacuolations (thin black arrows) in the neurons in the telencephalon. (C2) Micrographs of HE staining and ISH for muscle. Note the infiltration of blood cell (thin red arrows) in the abdominal muscles. (C3) Micrographs of HE staining and ISH for ovary. **D** The CMNV reverse transcription nested PCR assay results of the offspring fish individuals from the marine medaka parent of the viral infection group. **E** ISH analysis for CMNV-positive offspring fish with spinal curvature. E1–E3 indicate the positions of the vertebra, spinal cord and muscle, respectively. (E1) Micrographs of HE staining and ISH for vertebra. Note the vacuolations (thin black arrows) in vertebra. (E2) Micrographs of HE staining and ISH for spinal cord. Note the vacuolations (thin black arrows) in the spinal cord. (E3) Micrographs of HE staining and ISH for muscle. Note the muscle fragmentation with a tendency toward muscle lysis (thick black arrows) in the abdominal muscle. HE, H&E stained; ISH, in situ hybridization; Mag + , high magnification. Scale bars = 100 µm and 20 µm for low and high magnification, respectively

Towards further assessing the potential risk of infection to fish that consume krill harboring CMNV and PvPV, a portion of the Antarctic krill samples used for metatranscription analysis was fed to marine medaka *Oryzias melastigma*, a fish species commonly used in experiments. Individuals that were fed the virus-positive Antarctic krill did not show obvious clinical symptoms other than slower swimming and foraging when compared to the control group. However, 1/12 of the progeny from these fish did develop spinal curvature (Fig. 4A, Supplementary Fig. S3).

Notably, all parental fish that were fed with virus-positive krill were tested positive for CMNV via RT-qPCR, but negative for PvPV, SCRV and HSHRV. In contrast, CMNV was not detected in any individuals from the control group (Fig. 4B). In CMNV exposed group, viral loads increased slightly by day four post-exposure, followed by slight decreases over the next four days then increased substantially by 14 days post-exposure, reaching 674.6 virus copies/mg (Fig. 4B). Histopathological analysis of the fish from the infection group revealed severe vacuolation within the neurons of the telencephalon (Fig. 4C1), as well as lytic necrosis, hemorrhage and an inflammatory infiltrate within the abdominal muscles, where lytic necrosis was also present (Fig. 4C2). Concurrently, strong positive CMNV hybridization signals were detected by ISH in these same lesion areas (Fig. 4C1, C2), as well as in the ovaries (Fig. 4C3). In contrast, no CMNV hybridization signals were present in marine medaka from the control group (Supplementary Fig. S4).

Using both a CMNV RT-nPCR assay and ISH, 41.7% (5/12) of the progeny fish from the infection group were determined to be CMNV positive. In the infected group, 8.3% (1/12) CMNV-positive individuals showed spinal curvature, 33.3% (4/12) CMNV-positive individuals appeared normal, and the remaining 58.3% (7/12) CMNV-negative individuals were all normal (Fig. 4A, D, E). Similar as the parental adults, severe vacuolation was once again observed in the neurons of the telencephalon, vertebra and spinal cord (Fig. 4E1, E2), as was lytic necrosis within the abdominal muscle (Fig. 4E3). Concurrently, CMNV positive hybridization signals were present within these lesions (Fig. 4E1–E3).

**Fig. 5:**
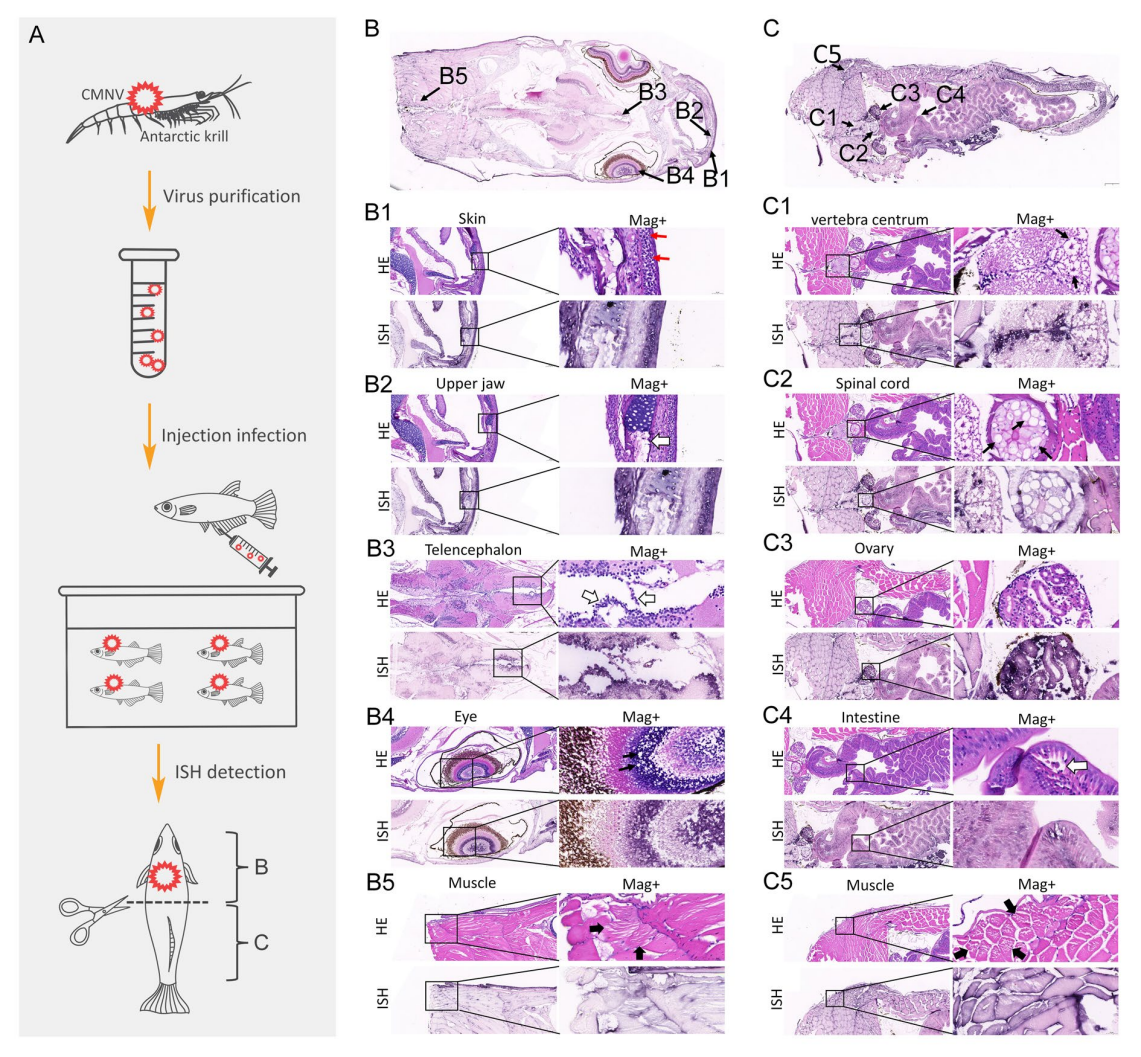
The schema and results of the test for infectivity and pathogenicity of CMNV_Es to marine medaka *Oryzias melastigma* **A** Schematic diagram of the experimental procedure. **B** and **C** indicate the positions of the figures (**B**) and figure (**C**), respectively. **B** H&E and ISH analysis of the head of marine medaka infected with CMNV by intraperitoneal injection. B1–B5 indicate the positions of skin, upper jaw, telencephalon, eye, and muscle, respectively. (B1) Micrographs of HE staining and ISH for skin. Note the karyopyknosis (thin red arrows) in the keratinocytes. (B2) Micrographs of HE staining and ISH for upper jaw. Note the shedding of chondrocyte of upper jaw (thick white arrows). (B3) Micrographs of HE staining and ISH for the telencephalon. Note the shedding of mitral cell from the telencephalon (thick white arrows). (B4) Micrographs of HE staining and ISH for the eye. Note the vacuolation (thin black arrows) in the bipolar cell layer of the eyes. (B5) Micrographs of HE staining and ISH for muscle. Note the dissolved necrosis of muscle (fat black arrows). **C** ISH analysis of the abdomen of marine medaka infected with CMNV by intraperitoneal injection. C1–C5 indicate the positions of the vertebra centrum, spinal cord, ovary, intestine and muscle, respectively. (C1) Micrographs of HE staining and ISH for vertebra centrum. Note the vacuolation (thin black arrows) in the vertebra centrum. (C2) Micrographs of HE staining and ISH for spinal cord. Note the vacuolation (thin black arrows) in the spinal cord. (C3) Micrographs of HE staining and ISH for ovary. (C4) Micrographs of HE staining and ISH for intestine. Note the shedding of columnar-shaped absorptive enterocytes of the intestine (thick white arrows). (C5) Micrographs of HE staining and ISH for muscle. Note the dissolved necrosis of muscle (fat black arrows). HE, H&E stained; ISH, in situ hybridization; Mag + , high magnification. Scale bar = 100 µm and 20 µm for low and high magnification, respectively

CMNV is the second most abundant virus in the Antarctic krill virome, and it has been reported to cause severe disease in farmed shrimp and fish. To exclude the possible interference of other viruses that were present in Antarctic krill in the assessment of the pathogenicity of CMNV_Es, CMNV was purified from Antarctic krill samples and further artificial infection experiments were conducted via intraperitoneal injection into healthy marine medaka (Fig. 5A). Individuals in the CMNV-injection group developed similar clinical symptoms as those observed in the medaka that were fed with CMNV_Es positive Antarctic krill, including swimming slowly. Likewise, karyorrhexis and pyknosis in the skin epidermal cells (Fig. 5B1), necrosis and sloughing of chondrocytes of the upper jaw, mitral cells of the telencephalon, and columnar absorptive enterocytes of the intestine (Fig. 5B2, B3, C4), vacuolization in the bipolar cell layer of the eyes, vertebral centrum, and spinal cord (Fig. 5B4, C1, C2), severe lytic necrosis of muscle (Fig. 5B5, C5), and edema within the ovary (Fig. 5C3) were observed in CMNV-injection-infected marine medaka. In all cases, CMNV positive hybridization signals were concurrently observed in association with these pathological changes (Fig. 5B, B1–B5, C, C1–C5).

## Discussion

Antarctic krill, one of the most abundant natural biological resources on earth, plays a critical role in the Antarctic food web and ecosystem stability (Cao et al. 2020; Wille et al. 2020). Unfortunately, little is known about the viruses associated with this invertebrate species. Herein, the first investigation into the composition and taxonomic diversity of viruses in Antarctic krill has been performed in the present study, which try to reveal the potential risk of the dominant viruses.

An in-depth examination of the metatranscription data related to virus gene sequence enrichment showed that 79.59% of the unigenes in the Antarctic krill virome were related to nine invertebrate viruses, most of which had low identities at the amino acid level with counterparts in the databases except for those related to PvPV and CMNV, also indicating that most of the related viruses were potentially novel. Among the viruses species of Antarctic krill virome that had similarities to the vertebrate viruses in GenBank, HSHRV and SCRV (Family *Rhabdoviridae*) were the two species with relatively high abundance. Interestingly, both of these viruses were originally isolated from diseased farmed fish (Liu et al. 2019; Ou et al. 2013; Tao et al. 2007; Zeng et al. 2014). Rhabdoviruses have single-stranded, negative-sense RNA genomes and are known for their broad host range that included invertebrates, vertebrates and plants (Dietzgen et al. 2020; Hause et al. 2020). So far, however, there have been no reports of HSHRV and SCRV infections in crustacea. The presence of suspected rhabdoviruses in the Antarctic krill virome provides new evidence for the further extended host spectrum of rhabdoviruses. Except vertebrate virus-related unigenes, mycovirus-related and protozoan virus-related unigenes were also found in the Antarctic krill virome. This may be due to the sample type used in the present study, i.e. the whole Antarctic krill individuals were homogenized for total RNA preparation and the samples included the unintended intestine contents.

The present study demonstrated that PvPV was the most abundant RNA virus in Antarctic krill, with a high infection rate in samples collected from the main fishing grounds in the Antarctic Peninsula region, western Antarctic during 2017–2019. Importantly, there was a significant increase in the prevalence of PvPV in Antarctic krill from 10.00 to 15.00% during this period. Notably, histopathological and ISH analyses revealed a clear association between the presence of PvPV and tissue damage in the host krill. Originally known as Wenzhou shrimp virus 8, PvPV was initially identified by transcriptome sequencing of coastal shrimp; however, its potential pathogenicity remained unexplored (Liu et al. 2021; Shi et al. 2016). In our study, we found that white leg shrimp exposed to PvPV exhibited gross and microscopic signs of disease, resulting in a cumulative mortality rate of 66.7% within 120 h. Furthermore, all infected individuals showed obvious pathological tissue damage in their muscle, hepatopancreas, eye and nerves, as well as strong signals of virus presence detected by ISH analyses. These results provide compelling evidence that PvPV_Es not only infects *P. vannamei* but also induces disease and mortality rates in them, confirming it as a pathogenic agent for shrimp species. Therefore, it is plausible that PvPV may represent an emerging threat to Antarctic krill, a matter that merits further comprehensive investigation.

CMNV, another virus previously reported in diseased shrimp, was also one of the high abundance viruses in the Antarctic krill virome. The prevalence of CMNV in the local Antarctic krill populations was much higher than the prevalence of this virus in both cultured crustaceans and coastal wild populations of *Larimichthys polyactis* (Li et al. 2019a, b; Xu et al. 2021). The prevalence of CMNV in Antarctic krill averaged over 75% during 2017–2019, and reaching 100% in 2017. This suggests that the virus is widespread in krill populations in the main fishery operation area of the Atlantic Ocean near the Antarctica. Conceivably, the virus may have been prevalent in the Antarctic krill for quite a period. CMNV, well-known for its wide host range, can infect marine and brackish shrimps, as well as invertebrates inhabiting shrimp farming ponds, and has caused severe economic losses of farmed shrimp in Southeast Asia and China (Pooljun et al. 2016; Thitamadee et al. 2016; Zhang et al. 2014).

Recent research has shown that CMNV is capable of crossing species barriers to infect and cause disease in a variety of teleost fish, including crucian carp (*Carassius auratus*), gobiid fish (*Mugilogobius abei*), Japanese flounder (*Paralichthys olivaceus*), zebrafish (*Danio rerio*) and small yellow croaker (*Larimichthys polyactis*) (Wang et al. 2019a,b, 2021; Xu et al. 2021; Zhang et al. 2018). As a key species in the Antarctic ecosystem, Antarctic krill infected by CMNV poses a potential ecological risk to predators, such as fish in Antarctic waters. The present study showed that the marine model fish medaka was infected by CMNV after feeding CMNV_Es-positive Antarctic krill. CMNV may infects its offspring by the way of vertical transmission. As the symptoms developed, the swimming and feeding activities of the parent fish decreased significantly, and the offspring showed severe tissue damage and spinal curvature. These results, together with evidence of natural CMNV-infections in krill predators (e.g., the electron sub-Antarctic lanternfish and the colossal squid), provide further evidence of the risk of CMNV transmission in the Antarctic food web, whether by horizontal transmission of CMNV-positive Antarctic krill being ingested by its predators, or by via transmission of CMNV from infected parents to their offspring. Moreover, the histological and ISH results showed that CMNV may infect multiple organs of both Antarctic krill and fish, where the virus was associated with a range of tissue damage. Overall, the present study reveals for the first time that CMNV may well affect the health of different fish species that feed on Antarctic krill in marine ecosystems. However, the temperature in Antarctica is low, and it is not known whether CMNV infection does harm seriously to its predators living in extremely low temperature water in the Southern Ocean.

With the development of commercial fisheries, Antarctic krill has become an important raw material resource for the aquaculture feed industry. Antarctic krill is sold as whole frozen krill in many scenarios for direct use as aquaculture feed, and as bait for sport fishing. Almost all krill catches in Canada are used as aquaculture feed, and fresh frozen krill in Japan is used directly as bait for fishing, and as food for fish farming (Wu and Xie 2010). In this context, Antarctic krill has become an important feed for many farmed aquatic animals at different life stages, including chum salmon (*Oncorhynchus keta*), rainbow trout (*Oncorhynchus mykiss*), common carp (*Cyprinus carpio*), red sea bream (*Pagrus major*), Japanese eel (*Anguilla japonica*), gray mullet (*Mugil cephalus*) and yellowtail (*Seriola quinqueradiata*) (Akiyama 1984; Allahpichay 1984; Yoshitomi and Nagano 2012). While exposure of krill to the high temperature of processing may inactivate the pathogens, the use of raw krill carries a high risk of introducing pathogens into aquaculture. The two emerging epidemics affecting the shrimp industry, Acute Hepatopancreatic Necrosis Disease (AHPND) and *Enterocytozoon hepatopenaei* (EHP), have been found that could be introduced into shrimp aquaculture through the use of fresh feed (Subasinghe et al. 2023).

More worryingly, previous studies suggested that viruses from the original environments of reservoirs may only pose a lower risk of pathogenicity to their original host because they are restricted by environmental factors and host factors (Letko et al. 2020; Lloyd-Smith 2017; Plowright et al. 2015). However, if they spill over from the original host or environment, they may represent an unprecedented risk under certain favorable conditions. In this context and because Antarctic krill live in cold marine waters ~ 0.64 to 1.32 °C year round, many viruses in the Antarctic krill virome, including PvPV and CMNV, may remain relatively inactive and not show high pathogenicity to their host. However, and if these viruses enter hosts living in ecological environments with higher temperatures, they may show higher pathogenicity and ecological risk. Indeed, previous reports have shown that CMNV is more pathogenic to farmed shrimp, especially *P. vannamei,* at higher temperatures (above 28 °C) (Zhang et al. 2014).

In summary, the present study revealed for the first time the composition and taxonomic diversity of viruses in Antarctic krill. Indeed, the two most abundant RNA viruses (e.g., PvPV and CMNV) were further shown to represent potentially new pathogenic and ecological risks to Antarctic krill and the animals that feed upon them in both culture ecosystems and wild Antarctic ecosystems. Moreover, findings suggest that Antarctic krill may represent an important virus reservoir in an extreme environment. That is, Antarctic krill may represent a new Pandora's box releasing new viruses with potential risk when its frozen product is used directly as feed for aquaculture, as well as bait for fishing.

## Supplementary Information

Below is the link to the electronic supplementary material.

Supplementary file1 (DOCX 1637 KB)

## Data Availability

Data available on request from the authors.
