# Supplementary material for: RNA virus diversity highlights the potential biosecurity threat posed by Antarctic krill
Source: Mar Life Sci Technol. 2025 Feb 13;7(1):96–109. doi: 10.1007/s42995-024-00270-w (PMC11871207; doi:10.1007/s42995-024-00270-w)
Supplement: Supplementary file 1 — Supplementary file1 (DOCX 1637 KB) [file 42995_2024_270_MOESM1_ESM.docx]

**Supporting information for**

RNA virus diversity highlights the potential biosecurity threat posed by Antarctic krill

Tingting Xu^1,2^, Xianyong Zhao^1,2^, Thomas Loch^3^, Jiancheng Zhu^1,2^, Wei Wang^1,2^, Xinliang Wang^1,2^, Cong Wang^1,2^, Gangzhou Fan^1,2^, Bin Hao^4^, Jichang Zhang^1,2^, Wenxiu Zhao^1,2^, Melba G. Bondad-Reantaso^4^, Victoria Alday-Sanz^5^, Qingli Zhang^1,2^*

*Corresponding author at:

Qingli Zhang

PhD, Professor

Yellow Sea Fisheries Research Institute Chinese Academy of Fishery Sciences, Qingdao 266071, China

Email: zhangql@ysfri.ac.cn

**This file includes:**

Supporting text

Figures S1 to S4

Tables S1 to S4


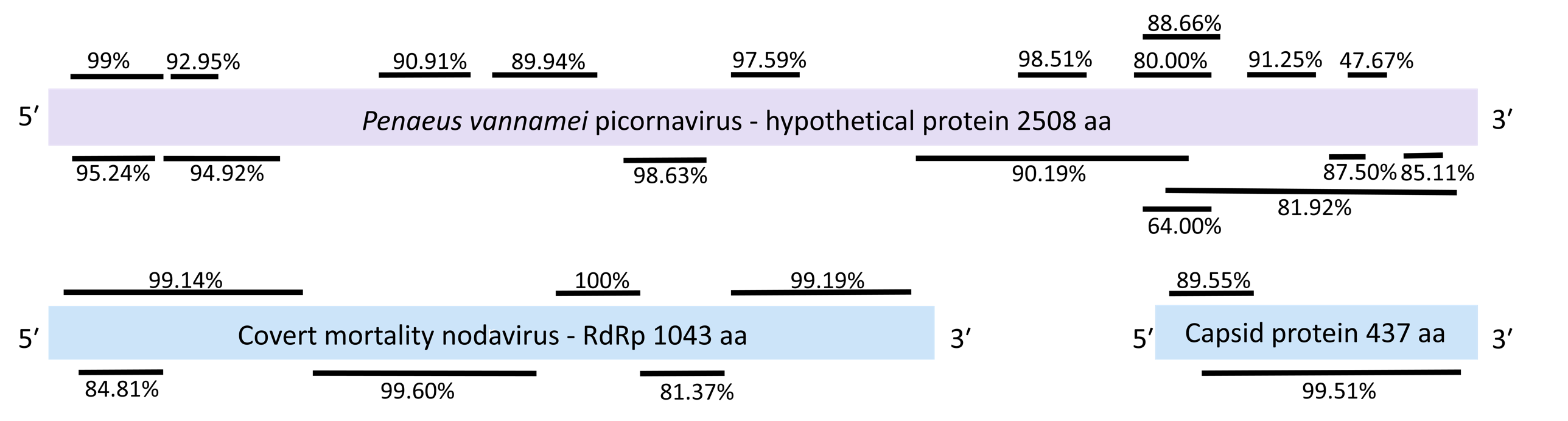


Fig. S1.

Sequence similarity and the positions of the amino acid sequence of the unigenes with those of known viruses.

**
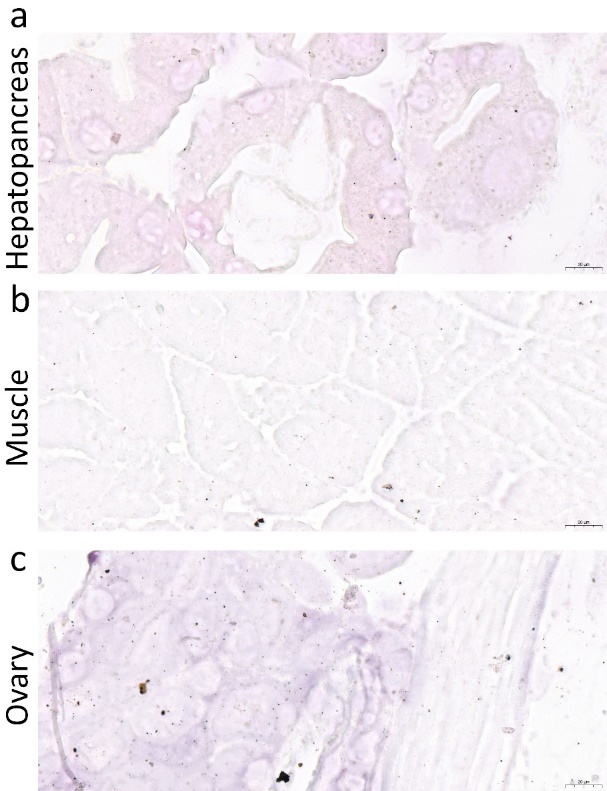
**

Fig. S2.

Micrographs of *in situ* RNA hybridization for muscle and hepatopancreas of both CMNV and PvPV positive Antarctic krill *Euphausia superba* with random probe. (a) hepatopancreas. (b) muscle. (c) ovary. Scale bars = 20 µm.


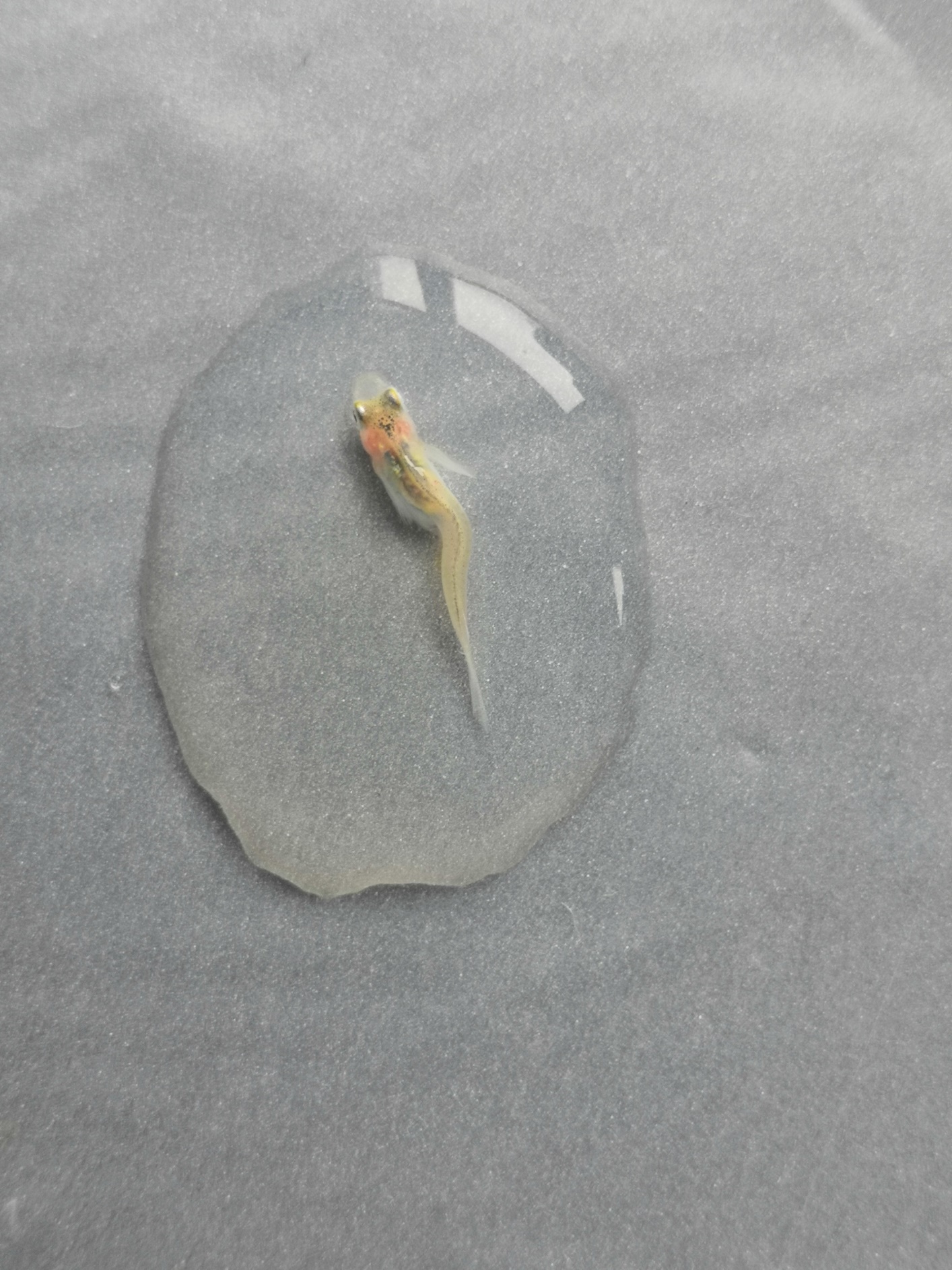


Fig. S3.

Offspring fish with spinal curvature of marine medaka *Oryzias melastigma* artificially fed with CMNV-positive Antarctic krill.


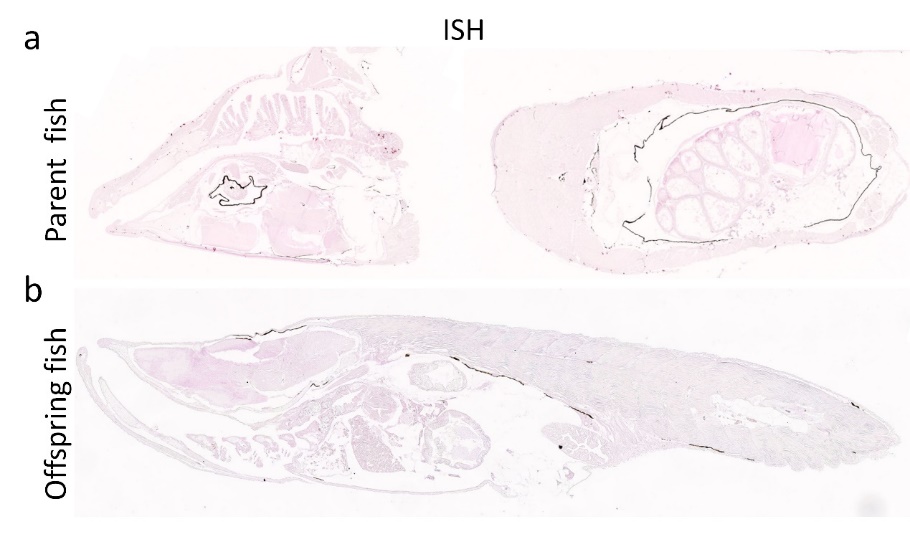


Fig. S4.

Micrographs of *in situ* RNA hybridization for parent marine medaka *Oryzias melastigma* and offspring marine medaka in control group. (a)Parent fish. Scale bars = 500 µm. (b) Offspring fish. Scale bars=200 µm.

Table S1.

Summary of sequenced data

| No. Raw Datas | No. Clean Reads | No. Adapter | No. Low Quality | No. viral  Reads | No. total Unigenes | Average  length | No. viral  Unigenes |
| --- | --- | --- | --- | --- | --- | --- | --- |
| 112,773,412 | 100,492,256 (89.11%) | 101,464 (0.09%) | 12,179,692 (10.80%) | 222,088 (0.22%) | 12,558 | 357 bp | 49 (0.39%) |

Table S2.

GenBank accession number of unigenes-related viruses in the Antarctic krill virome

| **Virus** | **Genbank No.** |
| --- | --- |
| *Penaeus vannamei* picornavirus | YP_009336733 |
| Covert mortality nodavirus | MT270124 |
| Wenzhou shrimp virus 9 | YP_009337869 |
| *Marsupenaeus japonicus* endogenous nimavirus | GBG35428 |
| Beihai mantis shrimp virus 6 | YP_009333376 |
| Wuhan Millipede virus 4 | APG78320 |
| *Lymantria dispar* multiple nucleopolyhedrovirus | AOW42758 |
| Dicistroviridae sp. | QDH87920 |
| Wuhan cricket virus 2 | YP_009345134 |
| Hybrid snakehead virus | AGI97138 |
| *Siniperca chuatsi* rhabdovirus | YP_802941 |
| Cedratvirus lausannensis | SOB74033.1 |
| Riboviria sp. | QDH87920 |
| Cedratvirus Zaza IHUMI | SPN79770 |
| Plasmopara viticola lesion associated Partitivirus 7 | QHD64796 |
| Rosellinia necatrix partitivirus 21 | BBU59850 |
| Bremia lactucae associated partitivirus 1 | QIP68017 |

Table S3.

Virus abbreviations and GenBank accession numbers in phylogenetic tree analysis

| **Virus** | **Abbreviation** | **Genbank No.** |
| --- | --- | --- |
| ***Nodavirus*** |  |  |
| Covert mortality nodavirus | CMNV-ES | OL310822 |
| Covert mortality nodavirus | CMNV-PV | MT270124 |
| Flock House virus | FHV | NP_689444 |
| Black beetle virus | BBV | YP_053043 |
| *Macrobrachium rosenbergii* nodavirus_China strain | MrNV China | AAQ54758 |
| *Macrobrachium rosenbergii* nodavirus_Australia strain | MrNV Australia | AEY63648 |
| *Macrobrachium rosenbergii* nodavirus_Malaysia strain | MrNV Malaysia | AEQ39078 |
| *Penaeus vannamei* nodavirus | PvNV | YP_004207810 |
| *Drosophila melanogaster* American nodavirus | DmANV | ACU32794 |
| Nodamura virus | NoV | NP_077730 |
| Boolarra virus | BoV | NP_689439 |
| Pariacoto virus | PaV | NP_620109 |
| Striped jack nervous necrosis virus | SJNNV | NP_599247 |
| Tiger puffer nervous necrosis virus | TPNNV | YP_003288759 |
| Atlantic halibut nodavirus | AHNV | AAY34458 |
| Golden pompano nervous necrosis virus | GPNNV | ACX54065 |
| Atlantic cod nodavirus | ACNV | ABR23192 |
| Dragon grouper nervous necrosis virus | DGNNV | AAU85148 |
| Barfin flounder nervous necrosis virus | BFNNV | YP_003288756 |
| ANV-SW-2009a | ANV-SW-2009a | GQ342965 |
| Hubei NLV-23 | Hb-NLV-23 | KX883137 |
| Wenzhou NLV-6 | Wz NLV-6 | KX883260 |
| Red spotted grouper nervous necrosis virus | RGNNV | ACX69744 |
| ***Picornavirus*** |  |  |
| *Penaeus vannamei* picornavirus | PvPV-ES | PQ442309 |
| *Penaeus vannamei* picornavirus | PvPV-PV | UIU06302 |
| Wenzhou shrimp virus 8 | WzSV8 | YP_009336733 |
| Picornavirales sp. | PV sp. | QYV43043 |
| Hubei picorna-like virus 49 | HbPV 49 | YP_009336567 |
| Picornavirales sp.2 | PV sp.2 | QKN88960 |
| Hubei picorna-like virus 51 | HbPV 51 | YP_009337724 |
| Beihai picorna-like virus 61 | BhPV 61 | YP_009333444 |
| Beihai picorna-like virus 62 | BhPV 62 | YP_009330001 |
| Rhopalosiphum padi virus | RpPV | AWM98375 |
| Dicistroviridae sp. | DV sp. | QJI52009 |
| Flumine dicistrovirus 15 | FDV 15 | UQB76000 |
| Shahe picorna-like virus 11 | ShPV 11 | YP_009336882 |
| Culex dicistrovirus 2 | CDV 2 | AXQ04777 |
| Wenzhou picorna-like virus 36 | WzPV 36 | YP_009336970 |
| Dicistroviridae sp.2 | DV sp.2 | QJI52009 |
| Picornavirales sp.3 | PV sp.3 | UGO57387 |
| Feksystermes virus | FSV | QRW42904 |

Table S4.

The TaqMan RT-qPCR assay results of *Electrona carlsbergi* and *Mesonychoteuthis hamiltoni*

| **Sample #** | **RT-qPCR CMNV copy/µg total RNA** | **PvPV** | **SCRV** | **HSHRV** |
| --- | --- | --- | --- | --- |
| EC2018001 | - | - | - | - |
| EC2018002 | - | - | - | - |
| EC2018003 | - | - | - | - |
| EC2018004 | 5152.55 | - | - | - |
| EC2018005 | 864.31 | - | - | - |
| EC2018006 | 2233.87 | - | - | - |
| EC2018007 | - | - | - | - |
| EC2018008 | - | - | - | - |
| EC2018009 | - | - | - | - |

Note: “-” represent negative of the virus.
